# Supplementary material for: Complexome profiling of the Chlamydomonas psb28 mutant reveals TEF5 as an early PSII assembly factor
Source: Plant Cell. 2025 Mar 17;37(6):koaf055. doi: 10.1093/plcell/koaf055 (PMC12164590; doi:10.1093/plcell/koaf055)
Supplement: koaf055_Supplementary_Data [file koaf055_supplementary_data.zip › psb28_interactive.zip!/plots/intensity/qualityPlots/peptides/_Cre03.g179901.t1.1.html]

### Cre03.g179901.t1.1

Trivial name:   
SeqCoverage [median]: 7.40  
Mass: 52.97 kDa  
  
 

| Sequence | MissedCleavage | Mass [Da] | wtIntensity | muIntensity | Score |
| --- | --- | --- | --- | --- | --- |
| LAAAGEAAAAR | 0 | 970.52 | 607.62 | 397.58 | 97.43 |
| LLAPHAAEPAAADTLVR | 0 | 1714.94 | 0.00 | 1153.91 | 52.25 |
| LVEAVQTATAR | 0 | 1157.64 | 0.00 | 418.02 | 107.79 |
|  |  |  | 607.6 | 1969.5 |  |
